# Rab11 is Required for Maintenance of Cell Shape Via βPS Integrin Mediated Cell Adhesion in Drosophila

**Published:** 2012

**Authors:** Tanmay Bhuin, Jagat Kumar Roy

**Affiliations:** Cytogenetics Laboratory, Department of Zoology, Banaras Hindu University, Varanasi 221 005, India.

**Keywords:** Amnioserosa, cellular morphology, cuticle, dorsal closure, *Drosophila*, Rab11

## Abstract

In eukaryotes, vesicle trafficking is regulated by the small monomeric GTPases of the Rab protein family. Rab11, (a subfamily of the *Ypt/Rab *gene family) an evolutionarily conserved, ubiquitously expressed subfamily of small monomeric Rab GTPases, has been implicated in regulating vesicular trafficking through the recycling of endosomal compartment. In an earlier communication, we have shown that Rab11 is required for cell adhesion, maintenance of cell shape and actin-cytoskeleton organization during *Drosophila* wing development. Here, we report that Rab11 is required for the maintenance of cell shape via βPS integrin mediated cell adhesion. Cuticle preparations of the embryos, when Rab11 is over-expressed or activity of Rab11 is reduced via a double-stranded RNAi line, show dorsal open phenotypes. Immuno-fluorescence and immuno-histochemical analyses on embryos in the same genetic backgrounds also affect the localization of βPS integrins from the adhesion site of leading edge and amnioserosa cells during the dorsal closure stages of embryogenesis as well as the cellular morphology (cell shape) of the lateral epidermal cells.

Intra-cellular vesicle transport is a complex process mediated by a diverse array of proteins. A large group of small monomeric GTP-binding proteins; “Rabs forming a major class of Ras superfamily (1) are essential component of this process”. These proteins are associated with various exocytic and endocytic organelles, as well as vesicles that are coupled with these compartments. Rab proteins regulate vesicular trafficking pathways, behaving as membrane-associated molecular switches (2). *Rab11* is an evolutionary conserved ubiquitously expressed subfamily of the *Ypt/Rab* gene family and is associated with recycling of endosomes (RE). Multiple roles of *Rab11* have been characterized in *Drosophila *during development, differentiation and signaling pathways. Rab11 endosomes are involved in the regulation of vesicle exocytosis and membrane growth during cellularization, a special form of embryonic cleavage in *Drosophila *(3) and is also essential for the invagination of furrows during *Drosophila *embryonic cleavage (4). Previous studies have shown that Rab11 is required for the process of myoblast fusion (5) and in the development of nervous system during *Drosophila* embryogenesis (6).

Dorsal closure (DC) in *Drosophila* is an important morphogenetic event that takes place during stages 13-15 of embryogenesis. It covers the dorsal hole and establishes the dorsal ectoderm by stretching the lateral epithelial cells over the amnioserosa (7). This process relies strictly upon coordinated changes in cell shape by cell elongation, without cell recruitment or proliferation (8). In embryos, Rab11 localizes at significant levels both in the lateral epidermal cells and in the amnioserosa during dorsal closure. It has been shown that loss of Rab11 function leads to improper dorsal closure in the developing *Drosophila* embryo (9).

Integrins are one of the best characterized and major cell surface receptors which mediate cellular connections between the ligands in the extracellular matrix to the actin cytoskeleton. They are heterodimeric protein complexes composed of an α and a β subunit. They are involved in the attachment of cells with the extra cellular matrix and also for the transmission of various signals from the extracellular environment into various signaling pathways in cells (10, 11).

*Drosophila* integrins serve as prototype for the genetic studies of cell adhesion proteins during development. Various functions of integrins in *Drosophila* development have been extensively studied like maintaining the close apposition of wing surfaces during wing morphogenesis (12), attachment of embryonic muscle to epidermis and mediating the connections between lateral surface of amnioserosa cells and leading edge (LE) cells during embryonic dorsal closure (13). Evidences point to the fact that integrin-extracellular matrix interactions occur between the lateral surfaces of the amnioserosa cells and the LE cells that effectively mediates cell-cell adhesion. It has been seen that *Drosophila* embryos lacking βPS integrin have a hole in the dorsal epidermis due to the failure of proper dorsal closure (13). In a recent communication, we have shown that Rab11 is involved in the trafficking of βPS integrins during *Drosophila* wing development. The studies have also shown that alterations of Rab11 functions results in the failure of βPS integrins to reach the focal adhesions (14). Here, we report that *Rab11* affects the localization of βPS integrins from the adhesion site of leading edge and amnioserosa cells during the dorsal closure stages of embryogenesis and also changes cellular morphology/cell shape of the lateral epidermal (LAE) cells.

## Materials and Methods

**Fly stocks, genetics and lethality assay**

The following stocks were used: *UAS-Rab11*^RNAi ^(15); (gift from D Ready), *UAS-Rab1*^CA ^(16); (a gift of M. Gonzalez-Gaitan). Other stocks were from the Bloomington Stock Centre. In order to replace *CyO* balancers in *Rab11*^CA^ allele, reporter lacZ fused with the *CyO*, *CyO-ftz-lacZ* balancer was introduced.

To determine the phenotypes generated by over-expressing *Rab11*^CA^ or *Rab11*^RNAi^ in epithelial tissue the homozygous *GAL4* line of *prd-GAL4* was crossed to heterozygous *UAS-Rab11*^CA^*/CyO-ftz-lacZ* or homozygous *UAS-Rab11*^RNAi^ flies so that, in the first case, 50% of the embryos would be driven by *GAL4* line, whereas 100% embryos would be driven in this manner in the case of the *Rab11*^RNAi^ line. Because of the presence of lacZ reporter in the balancer chromosome, the trans-heterozygotes with *prd-GAL4* or without *prd-GAL4* could easily be segregated by staining with anti-β-galactosidase antibody. Thus, unstained embryos obtained by blue balancer selection or embryos containing dsRNA driven by *prd-GAL4* were further processed for antibody (different markers) staining. 

Homozygous virgins from *GAL4 *line were crossed to homozygous *UAS-Rab11*^RNAi^ flies, and heterozygous constitutively active *UAS-Rab11*^CA^*/+* (obtained after crossing with Oregon R flies) male flies and eggs/embryos from the different genotypes were collected on separate agar plates; the total number of embryos in each plate was counted. Unhatched embryos were counted after 25-30 hrs of egg laying (at 25°C), dechorionated and then observed for lethality. Unfertilized eggs were also counted and discarded while calculating embryonic lethality. In each case, at least 5000 embryos were counted. The percentage of embryonic lethality (EL) was calculated by using the following formulae: For homozygous* Rab11* allele (*Rab11*^RNAi^): EL = Dead embryos X100/Total no. of fertilized eggs.

For heterozygous *Rab11* allele (*Rab11*^CA ^): EL = Dead embryos/X 100/total no. of fertilised eggs/2 [Total no. of fertilized eggs = total no. of eggs-no. of unfertilized eggs] 

[Dead embryos = total no. of fertilized eggs-total no. of hatched embryos]

**Immunostaining of embryos**

Antibody staining was performed as described by Van Vactor and Kopczynski (17) with slight modifications. The primary antibodies used were as follows: mouse monoclonal anti-phosphotyrosine (Santa Crutz; 1:100), mouse anti-βPS integrin (CF6G11, DSHB; 1:10); alkaline-phosphatase-conjugated goat anti-rabbit IgG (Sigma; 1:1000). Secondary antibodies used were as follows: AF-488-conjugated goat anti-mouse, biotinylated goat anti-mouse IgG (Jackson Immunoresearch; 1:300). Biotinylated secondary antibodies were used in combination with the Vector Elite ABC kit (Vector Laboratories, Calif.) according to the manufacturer’s instructions. For improved signal intensity and colour contrast, the staining was developed by using 0.5 mg/ml DAB (3, 3-diaminobenzidine) and 0.02% H_2_O_2_ (hydrogen peroxide); 8% nickel chloride was used for further enhancement of the regular DAB procedure. Fluorescent whole-mount embryos were mounted in 70% glycerol in phosphate-buffered saline (PBS) supplemented with 15% DABCO (1,4-diazabicyclo-[2.2.2]-octane) and images were taken on a BioRad MRC laser scanning confocal microscope, whereas horseradish peroxidase (HRP)-stained embryos were mounted in 70% glycerol in PBS and examined and photographed under bright-field optics. Images were processed with BioRad software and/or Adobe Photoshop.

**Cuticle preparation from wild type and mutant embryos**

Wild type embryos were allowed for a synchronised egg laying. After 4 hrs, the embryos were collected and allowed to develop for 17-22 hrs, so that the cuticle layer is secreted. The embryos were then dechorionised, fixed and devitalinized in the same way as it was done for immunostaining. However after the final wash with methanol the embryos were suspended in glycerol + acetic acid (1:4) solution at 65^0^C, overnight. They were then mounted in Hoyer’s medium and again kept at 65^0^C, overnight. The cuticles were then visualized under a phase contrast microscope.

Mutant embryos were processed 48 hrs after collection from an overnight egg lying on an agar plate. The dead embryos were selected. They were then dechorionised and devitelinized in the same way. They were then suspended in glycerol and acetic acid (1:4) solution for overnight at 65^0^C, mounted in Hoyer’s medium and kept at 65^0^C for overnight. The cuticles were then visualized under a phase contrast microscope.

## Results

**Loss of Rab11 function leads to the disinteg-ration of embryonic cuticles**

**Fig 1:**
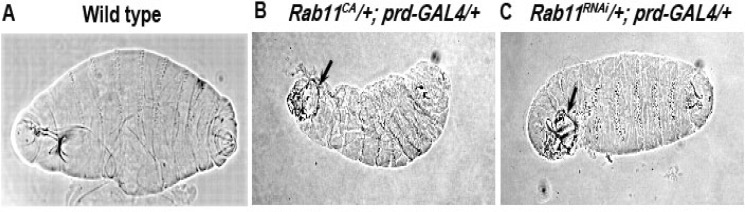
Cuticles of Rab11CA and Rab11RNAi mutant embryos displaying the presence of anterior/dorsal hole. Anterior is towards the left and the dorsal side is down in A, B and C. A, B and C show the cuticle of a wild type embryo and embryos from Rab11CA and Rab11RNAi, respectively. The black arrows in B and C indicate the anterior/dorsal hole

Studies have shown that βPS integrin is essential for proper dorsal closure to occur (13). The strongly hypomorphic/nearest to null allele of *Rab11* show defects in dorsal closure and thus have defective cuticle (9). It has also been shown that Rab11 is involved in the trafficking of βPS integrin during *Drosophila* wing development (14). Therefore, it has been hypothesized that there might be a loss of βPS integrin in the leading edge epidermal cells in *Rab11* mutant backgrounds. To test this in a tissue specific manner and to study the phenotypic consequences, the constitutively-active (*Rab11*^CA^) and the *Rab11*^RNAi^ alleles were expressed using UAS-GAL4 system (18) in the epidermal stripes driven by *prd-GAL4 *during dorsal closure stages of embryogenesis*. *Expression of these two *Rab11* transgenes with *prd-GAL4* causes embryonic to larval lethality (In case of *Rab11*^CA^ alleles; 12% embryonic and rest larval lethality whereas in case of *Rab11*^RNAi ^lines 10% embryonic and restlarval lethality were observed) which suggested further studies to analyse the epidermal development in these mutants. Cuticlepreparations of these lethal embryos were carried out which showed presence of dorsal/anterior openings (Fig. 1B and 1C) as compared to the wild type (Fig. 1A) in conformity with the previous observation (9).

**Inactivation of Rab11 function show loss of βPS integrins from the leading edge epidermal cells**

**Fig 2:**
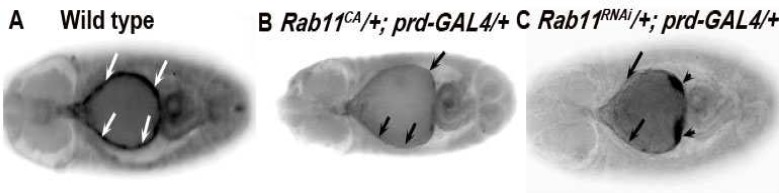
Immunostaining of stage 14 wild type (A), *Rab11*^CA^ (B) and *Rab11*^RNAi ^(C) embryos using anti-βPS-antibody. Anterior is on the left side in all the images. The arrows in B and C indicate the areas where there is a loss of βPS-integrins from the leading edge epidermal cells as compared with wild type. The arrowheads in C indicate the areas where there is an aggregation of βPS-integrins

To observe whether there is any significant loss of βPS integrin from the leading edge epidermal cells in Rab11 mutant backgrounds, immunostaining of whole embryos of *Rab11*^CA^ and *Rab11*^RNAi^ driven by *prd-GAL4* was carried out using anti-βPS integrin-antibody. It was seen that there was a significant (80 embryos, n=200) loss of βPS integrin in the leading edge epidermal cells of both the alleles (Fig. 2B and 2C) when compared to the normal wild type embryos (Fig. 2A). However in the *Rab11*^RNAi^ line aggregation of βPS integrin was also seen.

**Fig 3:**
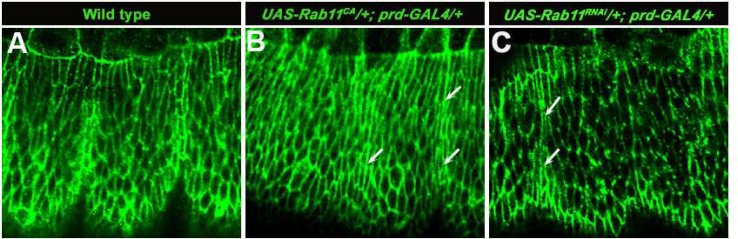
Confocal sections of wild type(A), *Rab11*^CA^(B) and *Rab11*^RNAi ^(C) embryos, respectively, stained with anti-phosphotyrosine antibody showing the distribution of tyrosine phosphorylated proteins in the leading edge and the lateral epidermal cells. The dorsal side is up in all the images. The arrows in B and C indicate the change of cell shape in the lateral epidermal cells when compared to the wild type embryo (A).

**Loss of Rab11 function results in the change of lateral epithelial cell shape**

It has been shown that integrin mediated cell-ECM adhesion is essential to maintain proper cell shape (12). We have recently shown that trafficking of βPS integrin through Rab11 endosomes is essential for cell adhesion, maintenance of cell shape and actin-cytoskeleton organisation during *Drosophila* wing development (14). To check the cell shape of the lateral epidermal cells in these two *Rab11* mutant backgrounds, immunostaining of the embryos was carried out using an apical marker, anti-phosphotyrosine antibody (19). This showed a change in the cell shape (Fig. 3B and 3C) when compared to the normal wild type embryos (Fig. 3A).

## Discussion

This study demonqstrates that in *Rab11* mutant background there is a loss of βPS-integrin from the leading edge cells and there is change in the cell shape of the lateral epidermal cells. Thus this shows that Rab11 is essential for the maintenance of βPS-integrin level in the leading edge epidermal cells and the proper maintenance of cell shape in the lateral epidermal cells. Previous studies in *Drosophila* wing development have shown that cell-ECM interactions mediated by integrins regulate the change in cell shape which is essential for morphogenesis (12). Since defects are seen in localisation of βPS-integrin in the leading edge cells of *Rab11* mutants, through this study we indirectly demonstrate that Rab11 is essential for the trafficking of βPS-integrin and thereby the proper maintenance of cell shape. This also supports the previous study done in *Drosophila *wing development (14).
